# Development and clinical performance of high throughput loop-mediated isothermal amplification for detection of malaria

**DOI:** 10.1371/journal.pone.0171126

**Published:** 2017-02-06

**Authors:** Rushini S. Perera, Xavier C. Ding, Frank Tully, James Oliver, Nigel Bright, David Bell, Peter L. Chiodini, Iveth J. Gonzalez, Spencer D. Polley

**Affiliations:** 1 Department of Clinical Parasitology, Hospital for Tropical Diseases, University College London Hospitals NHS Foundation Trust, Mortimer Market, London, United Kingdom; 2 FIND CH-1202 Geneva, Switzerland; 3 42 Technology Ltd., Cambridgeshire, United Kingdom; 4 Porvair Sciences Ltd, Norfolk, United Kingdom; 5 Global Good Fund, Intellectual Ventures Laboratory, Bellevue, Washington, United States of America; 6 London School of Hygiene and Tropical Medicine, Keppel Street, London, United Kingdom; 7 Department of Clinical Parasitology, Health Services Laboratories LLP, Hospital for Tropical Diseases, Mortimer Market, London, United Kingdom; Université Pierre et Marie Curie, FRANCE

## Abstract

**Background:**

Accurate and efficient detection of sub-microscopic malaria infections is crucial for enabling rapid treatment and interruption of transmission. Commercially available malaria LAMP kits have excellent diagnostic performance, though throughput is limited by the need to prepare samples individually. Here, we evaluate the clinical performance of a newly developed high throughput (HTP) sample processing system for use in conjunction with the Eiken malaria LAMP kit.

**Methods:**

The HTP system utilised dried blood spots (DBS) and liquid whole blood (WB), with parallel sample processing of 94 samples per run. The system was evaluated using 699 samples of known infection status pre-determined by gold standard nested PCR.

**Results:**

The sensitivity and specificity of WB-HTP-LAMP was 98.6% (95% CI, 95.7–100), and 99.7% (95% CI, 99.2–100); sensitivity of DBS-HTP-LAMP was 97.1% (95% CI, 93.1–100), and specificity 100% against PCR. At parasite densities greater or equal to 2 parasites/μL, WB and DBS HTP-LAMP showed 100% sensitivity and specificity against PCR. At densities less than 2 p/μL, WB-HTP-LAMP sensitivity was 88.9% (95% CI, 77.1–100) and specificity was 99.7% (95% CI, 99.2–100); sensitivity and specificity of DBS-HTP-LAMP was 77.8% (95% CI, 54.3–99.5) and 100% respectively.

**Conclusions:**

The HTP-LAMP system is a highly sensitive diagnostic test, with the potential to allow large scale population screening in malaria elimination campaigns.

## Introduction

Successful elimination of malaria is dependent on accurate and efficient diagnoses to actively treat and clear parasite infections, thereby interrupting transmission to new hosts. Microscopic examination of thick and thin blood smears remains a commonly used method for diagnosis of malarial infections [1], capable of detecting parasites in less than 60 minutes. Microscopy-based diagnosis is completely dependent upon the skill of trained microscopists and implementation of regular external quality assessment (EQA) schemes. The development of antigen-based rapid diagnostic tests (RDTs) allows for quick and accurate detection of infections [2]. Such testing is relied upon in areas lacking trained microscopists and specialised diagnostic equipment, and is increasingly being used for diagnostic testing over microscopy [3]. However, both techniques have limited sensitivity: the lowest detection limit is 5 to 50 parasites per microLitre (p/μL) of blood for expert microscopy, and around 100 p/μL for RDTs-, and therefore inadequate for detection of infections below these levels, which frequently occur [1, 3–6].

Malaria elimination programs require case management and community surveillance of asymptomatic infections which are sub-patent, i.e. where parasitaemia is below the threshold of detection by microscopy and RDTs, as such infections have proven capable of sustaining transmission of gametocytes to mosquitoes during blood meal feeds. Detecting such infections is particularly important during mass screen and treatment (MSAT) programs driving elimination efforts in these areas [7]. Therefore, highly sensitive molecular techniques for the detection of low density parasitaemia, such as nucleic acid amplification tests (NAATs), capable of detecting trace amounts of parasite DNA are needed.

Polymerase chain reaction (PCR) is one such test capable of amplifying low levels of parasite DNA. It allows highly sensitive detection of parasite DNA below the threshold of detection for microscopy and RDTs, making it the gold standard for detection of malarial parasitaemia [8, 9]. However, most PCR assays require specialised infrastructure with costly equipment and reagents, plus specialised training and results can take up to two days to generate from receipt of the sample, making it inappropriate to guide treatment. Loop mediated isothermal amplification (LAMP) is a molecular tool similar to PCR in that it amplifies parasite DNA with PCR level accuracy, whilst providing several advantages over PCR particularly for detection of infections in field settings [10, 11]. Amplification occurs in a single step under isothermal conditions using the *Bacillus stearothermophilus* polymerase enzyme, which does not require a high temperature denaturation step, or cyclical temperature changes, eliminating the requirement for expensive thermocyclers. Real-time detection can be performed using basic equipment i.e. a turbidimeter or visually using a UV light, compared to gel electrophoresis or real-time PCR machines required for PCR and qPCR respectively, and therefore suitable for laboratories lacking specialised infrastructure [12–14]. The method is also less time intensive with simpler sample processing requirements, and results can be obtained in under an hour, making it more suitable for field-based diagnostics. This has led to the development of two commercially available, CE marked, field-stable Loopamp^TM^ kits (Eiken Chemical Co.) for the detection of all *Plasmodium spp*. (Pan only kit) and *P*. *falciparum* specifically (Pan/Pf kit), which have been trialled in reference laboratory settings in UK and Europe, and in field settings in Uganda, Zanzibar and Colombia [10, 11, 15–19]. These amplification kits have been coupled with bespoke rapid DNA extraction technology (PURE), capable of extracting DNA without the need for pipetting or centrifugation. Alternatively, a simple boil and spin protocol (BS-LAMP) allows rapid extraction and amplification of DNA by LAMP [20].

While LAMP assays are capable of detecting parasite DNA in under an hour, throughput is restricted by time taken for sample processing and DNA extraction. The use of colorimetric indicators for visual detection of high throughput LAMP-amplified parasite DNA has been described [21–23]. Britton et al. describe a technique which uses 96 well plates for performing high throughput LAMP reactions using the dye hydroxynaphthol blue for a visual end point readout. However, the separate DNA extraction step is time-consuming with completion of the assay taking 4–6 hours. [21, 22]. Lucchi et al. describe a high throughput system utilising malachite green dye for visual assessment of LAMP products [23]. This system is compatible with the use of DNA extracted from whole blood as well as filter papers and showed a limit of detection between 1–8 p/μL. However, both colorimetric high throughput assays have separate sample preparation steps and utilise extemporaneously prepared LAMP reagents requiring additional pipetting steps as well as cold storage facilities and is not field adaptable when compared to stable kits with dried-down reagents.

The recently described Illumigene Malaria LAMP assays [24] use pre-dispensed, lyophilized reagents stable at ambient field conditions and employs either a simple filtration prep or a gravity-driven filtration prep for sample preparation. This system shows a turnaround time from sample preparation to reading results in less than an hour. The LAMP assay is performed and results analysed in a bespoke incubator/reader which allows testing of 10 samples per run thereby restricting the throughput of this assay [24].

Our study reports the development and performance evaluation of a novel high throughput (HTP) 96-well sample processing system for LAMP detection of malaria parasites, with turnaround from DNA extraction of 96 samples (94 samples and 2 controls) to reading results taking less than 2 hours. This system allows parallel processing of a large number of samples using a set of three inter-locking 96-well plates, with vacuum pressure transfer between plates, reducing the need for pipetting and eliminating centrifugation requirements. HTP-LAMP performance is evaluated using both dried blood spots on filter paper as well as liquid whole blood, where the samples are of known infection status as pre-determined by gold standard nested PCR (nPCR) performed prior to this study.

## Materials and methods

### Ethics

This study was awarded ethical clearance by the Research Ethics Committee of University College London Hospitals (REC reference 07/Q0505/60). The samples were collected as part of routine diagnostics and stored as fully anonymised samples surplus to diagnostic requirements. Therefore patient consent was not required.

### Samples and study site

The performance of the HTP-LAMP assay was characterized using a bank of 705 venous blood samples anticoagulated with either ethylenediaminetetraacetic acid (EDTA) or heparin, originally taken for routine diagnosis of malaria from patients of the Hospital for Tropical Diseases (HTD) London, from January to July 2011. Samples from sequential patients with a history of previous malarial infection or presenting with symptoms indicative of current malaria infection were tested within the Department of Clinical Parasitology at HTD for blood parasites using routine microscopy. The samples were anonymised and subsequently analysed by boil and spin LAMP (BS-LAMP) and PCR [18]. Excess anonymised samples from this study were stored at -20°C until their use for the evaluation of the high throughput sample processing system for LAMP.

The HTP-LAMP assays were performed by a single study researcher in the Department of Clinical Parasitology at HTD. The researcher did not have access to the database and was blinded to microscopy, PCR and BS-LAMP results.

### HTP sample processing

**Fig 1:**
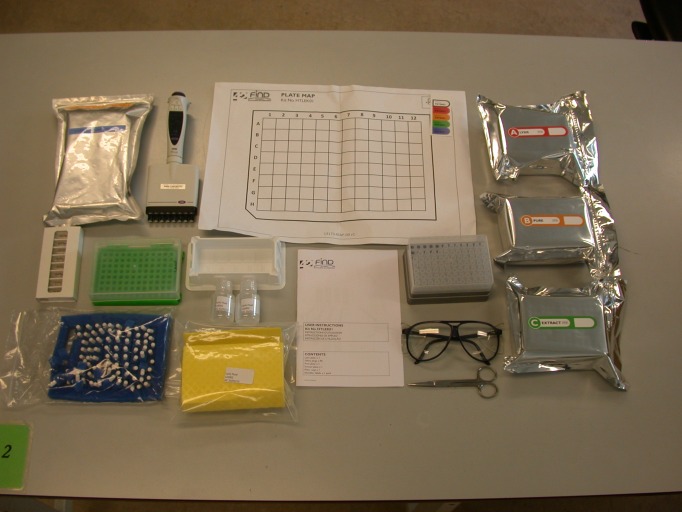
Layout of the contents of the high throughput sample processing kit on the bench. The contents of the sample processing kit which includes a LYSIS plate, PURE plate, EXTRACT plate, sample record sheet, lysis fluid, individual foam plugs and instruction manual.

**Fig 2:**
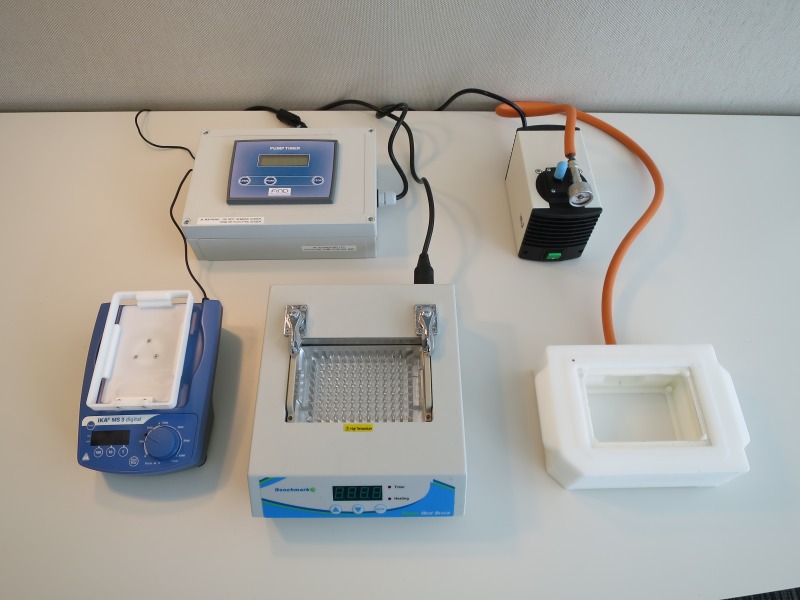
Layout of the high throughput set-up on the bench. The layout of the high throughput set-up which includes a shaker, hot block, vacuum manifold and pressure gauge.

**Fig 3:**
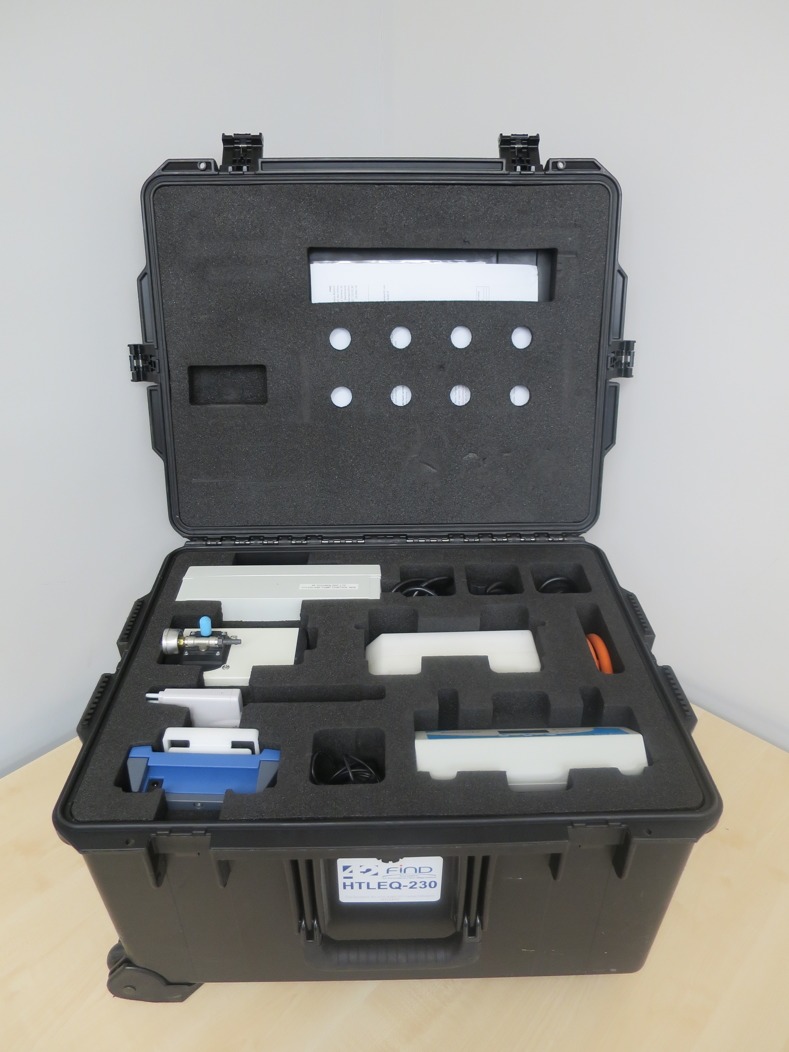
The contents of high throughput LAMP set-up packed into a transportable hard-cover case.

**Fig 4:**
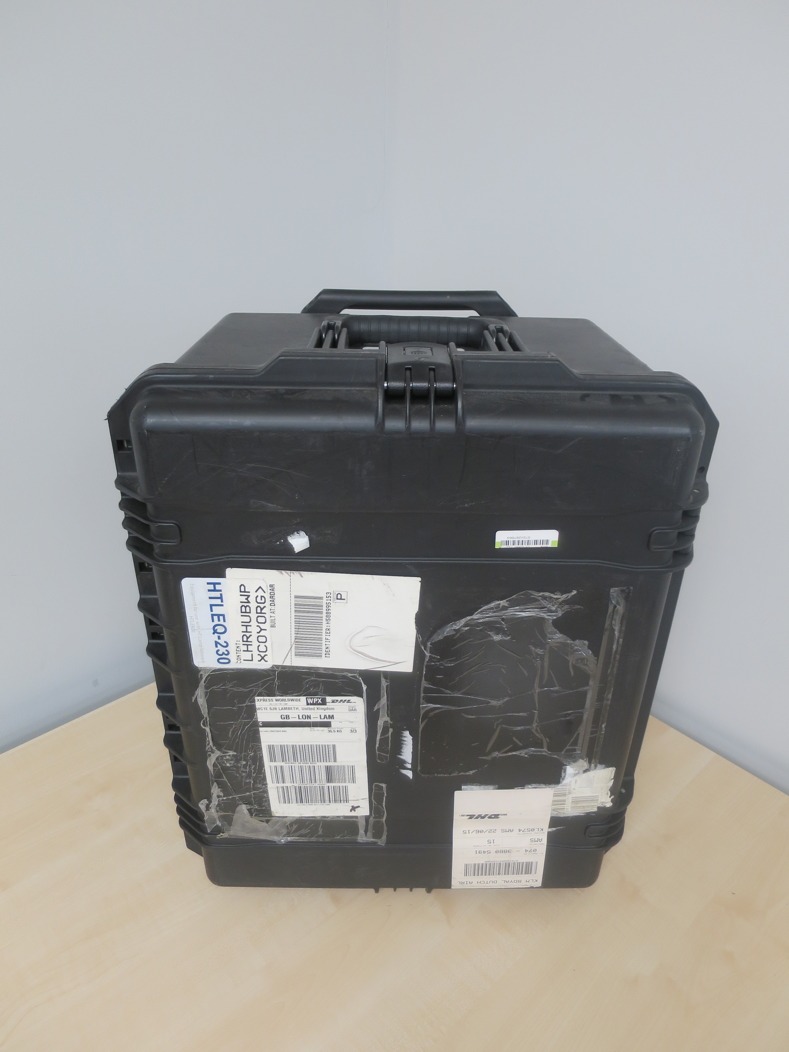
The transportable hard-cover case containing the high throughput LAMP set-up.

The HTP system was developed in association with 42 Technology Ltd., a commercial medical device development company, and Porvair Sciences, a company specialized in filtration plates, to convert the low throughput PURE extraction system into one that could process up to 96 samples within an hour. The extraction consumables kit includes a chart for recording the location of samples within the plate, three disposable 96 well plates for lysis, purification and elution and all reagents required (Fig 1). The hardware for this system consists of a heating block with bespoke machined insert for the blood lysis step, a shaker, a vacuum pump (with bespoke timer) and manifold to facilitate transfer of samples between plates (Fig 2). The system is “keyed” to prevent plates being inappropriately rotated in the system and ensure the provenance of each DNA extract. Each kit has a set of unique stickers to be affixed to each of the plates and the sample sheet to ensure there is no mix up where multiple batches of plates are to be processed. The whole system is designed to fit within a mobile flight case for transport (Figs 3 and 4) and to be run in laboratories/field stations with a steady power supply, but no other specialist equipment. LAMP amplification kits were purchased from Eiken Chemical Company (Japan).

Samples were tested as both whole blood (WB) and dried blood spots (DBS). The high throughput sample processing system was carried out in parallel batches of 96 samples consisting of 47 DBS samples, 47 WB samples, and *Plasmodium* positive and negative extraction controls.

The DBS samples were prepared by pipetting 20 μL of blood onto specifically designed shaped filter paper devices [25], which were then shut and placed in a zip-lock bag for 1 hour prior to DNA extraction to simulate sample collection and transport. The LYSIS plate was prepared by pipetting 498 μL of lysis fluid into each well using a multi-channel pipette. The filter paper sticks were cut a few millimetres above the unsoaked part directly into appropriate wells of the LYSIS plate, ensuring the scissors did not touch blood. For WB samples, 20 μL of WB was pipetted into appropriate wells of the LYSIS plate. Foam plugs were pushed down into each well and the LYSIS plate was placed in a shaker for 10 min at 750 revolutions per minute (rpm) to facilitate liberation of the blood and DNA from the filter papers. The LYSIS plate was placed in a hot block at 95°C for 14 min, then immediately removed, and placed on top of the vacuum manifold containing the PURE plate. Lysed samples were then transferred under vacuum pressure to the PURE plate. The PURE plate was placed in a shaker at 1000 rpm for 10 min, then placed on top of the vacuum manifold containing the EXTRACT plate. Purified DNA was transferred under vacuum pressure to the EXTRACT plate. Eluted DNA was used immediately in the LAMP reactions.

A multi-channel pipette was used to transfer 30 μL of DNA into Pan-LAMP (all species of *Plasmodium*) detection tubes. Tubes were inverted, incubated to allow dissolving of dried LAMP reagents in the tube caps, mixed by inversion, contents collected at the bottom of tubes, and incubation for amplification was performed immediately.

An instructional video demonstrating the standard operating procedure for the high throughput LAMP sample processing kit and LAMP amplification is available at https://youtu.be/95g0nMbaOGg

### LAMP amplification

Reaction tubes were incubated at 65°C for 40 min in a real time LA-320C turbidimeter (Eiken Chemical). Reactions were terminated by incubating at 80°C for 5 min. LAMP was performed in batches of 32 samples which included positive and negative control samples. The real time turbidity data were recorded, positives scored based on the first point at which the change in turbidity increased by 0.1 optical density (OD) units per second.

### BS-LAMP vs. HTP-LAMP

A comparison between the HTP-LAMP and BS-LAMP sample processing was performed using a tenfold dilution series of *P*. *falciparum* infected blood ranging from 10,000 parasites/μL to 0.1 p/μL (calibrated against the *Plasmodium falciparum* WHO International Standard) [26]. DNA was extracted *via* both boil and spin method ([20], S1 Text), and high throughput PURE method, and LAMP amplified in malaria pan detection tubes.

### Statistical analysis

A pairwise comparison for non-equivalence between reference (nPCR) and index test (WB-HTP-LAMP or DBS-HTP-LAMP) was determined by the McNemar test. The null hypothesis assuming no difference in performance between the reference test and index test was retained for *P* values ≥ 0.05. Statistical analysis was conducted using STATA 12.1 (Stata Corp, Texas, USA).

The study was performed in compliance with the updated version of Standards for Reporting of Diagnostic Accuracy (STARD) [27].

### Stability of PURE extracts

Stability of PURE extracts was tested using infected blood, PURE buffer and powder in the volumes used for the original PURE system. 35 μL of blood containing 0.2, 0.8, 2.0 or no parasites (neg) per microLitre was used in each extraction to produce over 180 μL of eluate in a 1.5 mL snap lock tube. The eluate was then aliquoted into 200 μL individual tubes and used immediately (0 hours) or after pre-defined incubations. Tubes were incubated at 24, 37 or 45 degrees C for various durations before use. The eluates were then added to Pan reaction tubes and run on a turbidimeter (S2 Fig).

## Results

### High throughput sample processing system development

The system was developed to allow the PURE technology to be re-engineered into a 96 well format. 96 well filter plates were tested to evaluate the optimum filter composition for fluid retention, DNA penetration and burst pressure. A bespoke manifold system was developed to allow keying of plates and easy operation of the system, whilst the optimum pump speed and pressure was also evaluated with a number of commercial systems. A hot air heating system for the LYSIS plate was replaced with a bespoke tooled aluminium heating block, which was tested by thermal mapping to ensure correct heating characteristics. The optimum PURE to lysis fluid ratios were evaluated to ensure cross contamination was minimised while maximising repeatability and sensitivity. Cross contamination was also minimised by the use of the drip control layer on the elution plate, a foam barrier on the bottom of the LYSIS plate, and packing prevention inserts in the PURE plate. The drip catcher was a bespoke moulded rubber system to ensure no leakage of hot, caustic lysis fluid. Numerous foam samples were evaluated to find one that would prevent leakage of lysis fluid in the event of a plate being dropped, whilst allowing thermal expansion in the gas layer to pass through so as not to increase the intra-well pressure during heating.

### High throughput sample processing system evaluation

**Fig 5:**
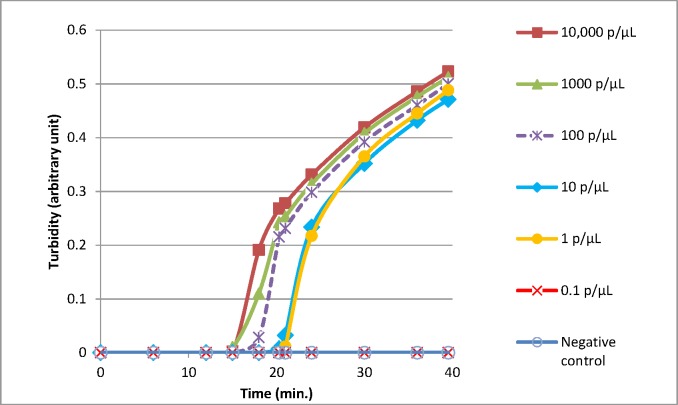
Limit of detection of LAMP reactions using the boil and spin DNA extraction method. Representative amplifications experiments using serial dilutions of samples of known parasitaemia (quantified using the WHO International Standard as calibrator).Quantification was achieved by extracting DNA from a parasite dilution series made in whole blood using a Qiacube and DNA blood mini kit (Qiagen, De Hilden). Extracted DNA was then quantified using the Shokoples real time PCR [28] against a within run standard curve created from DNA extracted from the WHO international standard using the same methodology. Parasite count per microLitre was calculated given an initial parasite burden of 10% in the WHO standard and an assumed red cell count of 5 x10^6^ red cells per microLitre. Mean time to turbidity in minutes as follows: 10,000 p/μL = 15.8; 1,000 p/μL = 15.9; 100 p/μL = 16.7; 10 p/μL = 18.2; 1 p/μL = 24.5.

**Fig 6:**
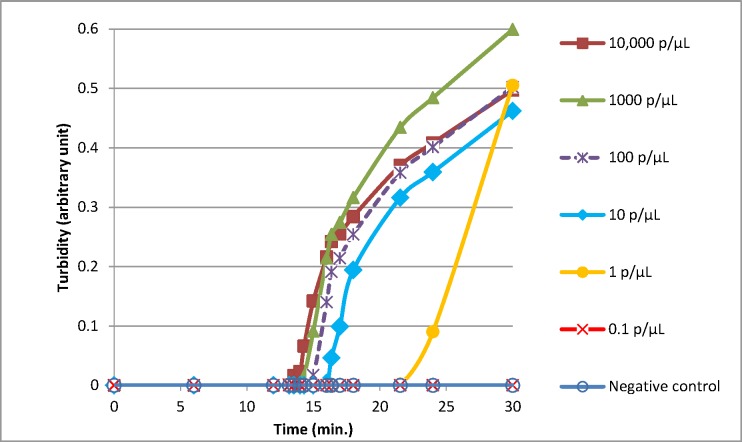
Limit of detection of LAMP reactions using the high throughput sample processing system. Representative amplifications experiments using serial dilutions of samples of known parasitaemia (quantified using the WHO International Standard as calibrator). Mean time to turbidity in minutes as follows: 10,000 p/μL = 17.5; 1,000 p/μL = 18.5; 100 p/μL = 19.4; 10 p/μL = 21.3; 1 p/μL = 22.9.

Comparison of the BS-LAMP versus the HTP-LAMP extraction was carried out using a range of dilutions of known *Plasmodium* positive blood samples. Figs 5 and 6 show the increase in turbidity with time measured for 10,000 p/μL to 0.1 p/μL for BS-LAMP and HTP-LAMP respectively. All samples from 10,000 p/μL to 1 p/μL amplified with both methods. The 1 p/μL sample reached turbidity at ~25 min for BS-LAMP and ~23 min for HTP-LAMP. The 0.1 p/μL sample did not amplify with either method, indicating a limit of detection of 1 p/μL. This was seen with all three repeats of the experiment.

### HTP-LAMP vs. PCR

**Fig 7:**
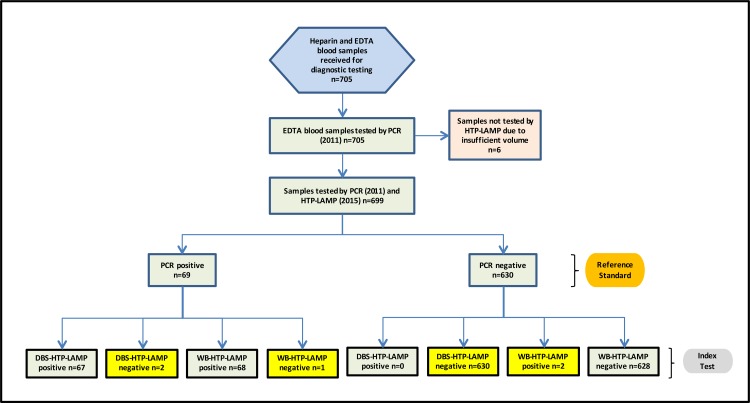
Flow chart of the study. The results of the two index tests, DBS-HTP-LAMP and WB-HTP-LAMP, against the gold standard nPCR results are summarized. Discrepancies between the gold standard and the index tests are highlighted. Abbreviation: EDTA ethylenediaminetetraacetic acid; PCR polymerase chain reaction; HTP-LAMP dried blood spot high throughput loop mediated isothermal amplification; DBS dried blood spot; WB whole blood.

From the 705 samples previously tested by PCR, microscopy and BS-LAMP for *Plasmodium* infection, 699 EDTA blood samples were available for use in the high throughput sample processing system evaluation, where DNA was extracted from both dried blood spots and whole blood. Six samples were excluded from this study due to insufficient material for testing in the original archived EDTA sample (Fig 7).

When clinical sensitivity and specificity was calculated for the HTP LAMP a similar diagnostic sensitivity and specificity to the reference 3-well nPCR was seen using both dried blood spots (DBS-HTP-LAMP) and whole blood (WB-HTP-LAMP) as the initial sample (Table 1).

**Table 1:** Comparison of Malaria HTP-LAMP results from 699 DBS and WB samples against the equivalent gold standard nPCR results from 2011.

Comparison	PCR(+)	PCR (-)	Sensitivity (95% CI)	Specificity (95% CI)	PPV, %	NPV, %
**DBS-HTP-LAMP vs. nPCRn = 699**						
DBS HTP-LAMP (+)	67	0	97.1	100	100	99.7
DBS HTP-LAMP (-)	2	630	(93.1–100)			
**WB-HTP-LAMP vs. nPCR n = 699**						
WB-HTP-LAMP (+)	68	2	98.6	99.7	97.1	99.8
WB-HTP-LAMP (-)	1	628	(95.7–100)	(99.2–100)		

The McNemar test, used to conduct a pairwise comparison between reference nested PCR and index text WB-HTP-LAMP, showed statistical equivalence between both tests (P = 0.5637), while the DBS-LAMP index test was found statistically non-inferior to the reference nPCR test (P = 0.157).

The WB-HTP-LAMP assay failed to detect 1 of 69 positive PCR samples, resulting in a sensitivity of 98.6% (95% CI, 95.7–100) for this assay (Table 1). This single false negative sample contained <1 p/μL according to the real time PCR analysis. HTP-LAMP therefore has a clinical sensitivity of 100% in samples with ≥1 p/μL when compared to 3-well nPCR. Two WB-HTP-LAMP positive samples were negative by nPCR, giving a specificity of 99.7% (95% CI, 99.2–100) (Table 1). Both samples showed late amplification, past 30 min. The positive predictive value (PPV) and negative predictive value (NPV) of WB-HTP-LAMP was 97.1% and 99.8% respectively, when compared with nPCR (Table 1). Superior results were seen when all samples <1 p/μL were excluded. A pairwise comparison between reference nPCR and the index text WB-HTP-LAMP showed statistical equivalence between both tests (P = 0.5637) (Table 1).

The DBS-HTP-LAMP assay failed to detect 2 of 69 positive PCR samples, resulting in a sensitivity of 97.1% (95% CI, 93.1–100) for this assay (Table 1). The two false negative samples contained <1 p/μL according to real time PCR analysis [18]. HTP-LAMP therefore has a clinical sensitivity of 100% in samples with ≥1 p/μL when compared to 3-well nPCR. The DBS-HTP-LAMP assay was 100% specific against nPCR (Table 1). Compared with nPCR, the PPV and NPV were 100% and 99.7% respectively for DBS-HTP-LAMP (Table 1). These results were seen when all samples were included in the analysis. Superior results were seen when all samples <1 p/μL were excluded. The DBS-LAMP index test was found to be statistically non-inferior to the reference nPCR test (P = 0.157) as calculated by the McNemar test (Table 1).

Analysis of the results following stratification of parasitaemia with a cut-off at 2 p/μL (WHO recommendation for performance of NAATs [29], shows a high level of diagnostic accuracy of the WB HTP-LAMP assay, similar to nPCR at ≥2 p/μL, with 100% sensitivity and specificity (Table 2). At < 2 p/μL, WB-HTP-LAMP showed diagnostic accuracy similar to nPCR with a sensitivity of 88.9% (95% CI, 77.1–100) and specificity of 99.7% (95% CI, 99.2–100) (Table 2).

**Table 2:** Stratified analysis of parasitaemia comparing DBS and WB HTP-LAMP against nPCR with a cut-off at 2 p/μL.

***Parasitaemia >2 p/μL***	**PCR(+)**	**PCR(-)**	**Sensitivity(95% CI)**	**Specificity(95% CI)**	**PPV, %**	**NPV,%**	**Parasites/μL^a^ (Geometric mean)**	***Plasmodium* species distribution**
**DBS-HTP-LAMP vs. nPCR n = 688**								
DBS HTP-LAMP (+)	60	0	100	100	100	100	2.0–912896.0	n = 57, *P*. *falciparum* from ≥ 4 p/μL
DBS HTP-LAMP (-)	0	628					(711.5)	n = 2, *P*. *vivax* each at 2 p/μL;n = 1, *P*. *ovale* at 2 p/μL
**WB-HTP-LAMP vs. nPCR n = 688**								
WB-HTP-LAMP (+)	60	0	100	100	100	100	2.0–912896.0	n = 57, *P*. *falciparum* from ≥ 4 p/μL
WB-HTP-LAMP (-)	0	628					(711.5)	n = 2, *P*. *vivax* each at 2 p/μL;n = 1, *P*. *ovale* at 2 p/μL
***Parasitaemia < 2p/μL***	**PCR (+)**	**PCR (-)**	**Sensitivity(95% CI)**	**Specificity (95% CI)**	**PPV, %**	**NPV, %**	**Parasites/μL**^**a**^ **(Geometric mean)**	***Plasmodium* species distribution**
**DBS-HTP-LAMP vs. nPCR n = 639**								
DBS HTP-LAMP (+)	7	0	77.8	100	100	99.7	undetected-1.5	n = 5, *P*. *falciparum* from 0.5–1.5 p/μL
DBS HTP-LAMP (-)	2	630	(54.3–100)				(1.03)	n = 2, *P*. *falciparum*, *P*. *ovale* (undetected by qPCR^b^, DBS positive) n = 1, *P*. *falciparum* at 0.8 p/μL,
								(DBS negative)
								n = 1, *P*. *falciparum* (undetected by qPCR^b^; DBS negative)
**WB-HTP-LAMP vs. nPCR n = 639**								
WB-HTP-LAMP (+)	8	2	88.9	99.7	80	99.8	undetected-1.5	n = 5, *P*. *falciparum* from 0.5–1.5 p/μL
WB-HTP-LAMP (-)	1	628	(71.1–100)	(99.2–100)			(1.03)	n = 2, *P*. *falciparum* (undetected by qPCR^b^, WB positive) n = 1, *P*. *ovale* (undetected by qPCR^b^, WB positive)n = 1, *P*. *falciparum* at 0.8 p/μL, (WB negative)

^a^ Range of parasite densities for PCR positive results

^b^ Parasite density could not be quantified due to negative qPCR results.

A stratified analysis of the DBS-HTP-LAMP assay shows a high level of diagnostic accuracy similar to nPCR at ≥ 2p/μL with 100% sensitivity and 100% specificity. The parasite densities in this group ranged from 2–912896 p/μL [18], with a geometric mean of 711.5 p/μL (Table 2).

At < 2 p/μL, DBS-HTP-LAMP showed a lower diagnostic accuracy compared to nPCR with a sensitivity of 77.8% (95% CI, 54.3–99.5) and 100% specificity (Table 2). The parasite densities in this group ranged from 1.5 p/μL to undetected *via* qPCR [18], with a geometric mean of 1.03 p/μL (Table 2).

## Discussion

Active identification and treatment of sub-patent infections in asymptomatic parasite carriers is crucial to achieving the World Health Organization (WHO) goal of 90% reduction in global malaria incidence and mortality rates by 2030 [30].

Early malaria elimination interventions in the 1950s-1970s involved mass drug administration (MDA) programmes where entire populations were administered therapeutic antimalarial drugs regardless of infection status, parasitaemia or symptoms with the aim of interrupting transmission. Whilst this strategy provided a means of treating asymptomatic carriers thereby reducing transmission rates, widespread issues regarding efficacy, resources, sustainability and increased drug resistance meant this approach was not feasible as a long term solution, though development of new antimalarial drugs may resolve some of these concerns [31].

As more countries move towards elimination, the importance of sub-microscopic reservoirs increases, thereby sustaining transmission from humans to mosquitoes. Recent studies have shown sub-microscopic infections are the source of 20–50% of human to mosquito infections in areas with low to very low transmission intensity, and are contributors to sustained transmission in these areas [6]. Detection of these reservoirs becomes increasingly important as control efforts move towards reduced transmission rates, and is key to interrupting transmission.

MSAT is aimed at screening large numbers of people to detect and treat anyone infected with malaria parasites. Furthermore, this allows the detection of carriers with low-density asymptomatic cases to also be identified and treated, thereby reducing transmission rates by targeting the human reservoir and interrupting transmission [32]. However, limitations of this strategy involve diagnostic accuracy and sensitivity of the screening method, where accurate and efficient detection requires a highly sensitive, field applicable molecular screening tool, with the capacity to diagnose large numbers of cases. The HTP-LAMP platform described in this paper offers the potential to be used as one such screening tool, and shows significant improvements and advantages over previously described versions of LAMP.

The HTP system accommodates complete processing and evaluation of up to 94 samples with controls in less than two hours. With two people performing this assay, double the number of samples can be tested, making it possible to scan 400 to 500 samples a day. Therefore, this system allows significantly faster turnaround time for processing large batches of samples compared to other diagnostic methods, including other high throughput LAMP kits ([21–24], Table C in S1 Text). In addition, DNA extracted using this system remains stable on the bench at room temperature for one day, facilitating repeat testing of positives from the same plate for species identification (S2 Fig).

With high throughput studies, the risk of incorrect sample loading or sample mix up is high. This system includes sample processing sheets, ID stickered plates specific to each kit to ensure correct loading of samples and easily identifiable plates which eliminates the risk of mixing up specimens or plates. The inter-locking plates are also designed to fit in one specific orientation which also minimises incorrect handling.

Use of dried blood spot filter paper devices has provided an efficient means of collecting and transporting large numbers of patient blood samples of defined volume ready for testing by molecular and serological methods. Prior studies have shown DNA extracted from these devices for malaria LAMP detection perform similar to DNA extracted from whole blood [33]. DNA extracted from DBS using a DNA blood mini kit (Qiagen Germany) has shown a detection limit of 1–2 p/μL [25] and the PURE DNA extraction method giving an LOD of 1.5 p/μL for DBS samples compared to 0.5 p/μL LOD of whole blood [11, 18]. Our results show that this relatively simple high throughput system for DNA extraction works as well using both DBS and WB samples with comparable sensitivity, detecting low density parasitaemias down to 1 p/μL, similar to the diagnostic accuracy of the gold standard PCR [1]. Given that previous studies have only been reported on Whatmann 3MM paper to date, it is unknown how papers other than 3MM or GB003 may perform with this system.

Utilisation of DBS specimens, for which only a pair of scissors is required for loading samples directly into the DNA extraction stage, provides an efficient means of processing large batches of samples and would be ideal for use in field environments. This system would be well suited for testing for infection using either source, a criterion particularly relevant during mass screening and field testing procedures and would be valuable in non-referral laboratory settings. The components of the HTP-LAMP kit fit on a small work bench and pack into an easily transportable case ready for deployment (Figs 3 and 4), thus making this system ideal for high throughput detection in remote field diagnostic labs, mobile screening stations and mass screening and treatment programmes.

Limitations of the current study involved a longer time to read results for all 96 samples due to the limited capacity of the 32 well turbidimeter. However, commercially available 96-well turbidimeters could be used to increase the throughput, allowing faster detection and quicker turnaround time from sample processing to reading results. Throughput could also be increased by the use of simple hot blocks with fluorescent calcein used for visual confirmation of positive results under a simple UV lamp. Other limitations of this study include the requirement of a stable power supply as well as maintenance of sterile conditions and the cost associated with the HTP set-up and LAMP reaction tubes.

Field trials using the HTP-LAMP system to test asymptomatic individuals have been performed in Zanzibar [34]. Further work will be needed fully to evaluate diagnostic sensitivity in field settings, particularly in areas with different malaria prevalence rates. The implementation of this robust and highly sensitive diagnostic system in endemic countries, particularly where resources are scarce and laboratory infrastructure lacking, would facilitate a more accurate means of detection of low-density parasite infections, particularly crucial for countries close to elimination. Such strategies would enable rapid treatment and proper surveillance required for successful elimination of malaria.

## Supporting information

S1 Fig (TIF)

S2 Fig (TIF)

S1 Text (DOCX)
