# Supplementary figures and images for: Development and clinical performance of high throughput loop-mediated isothermal amplification for detection of malaria
Source: PLoS One. 2017 Feb 6;12(2):e0171126. doi: 10.1371/journal.pone.0171126 (PMC5293265; doi:10.1371/journal.pone.0171126)

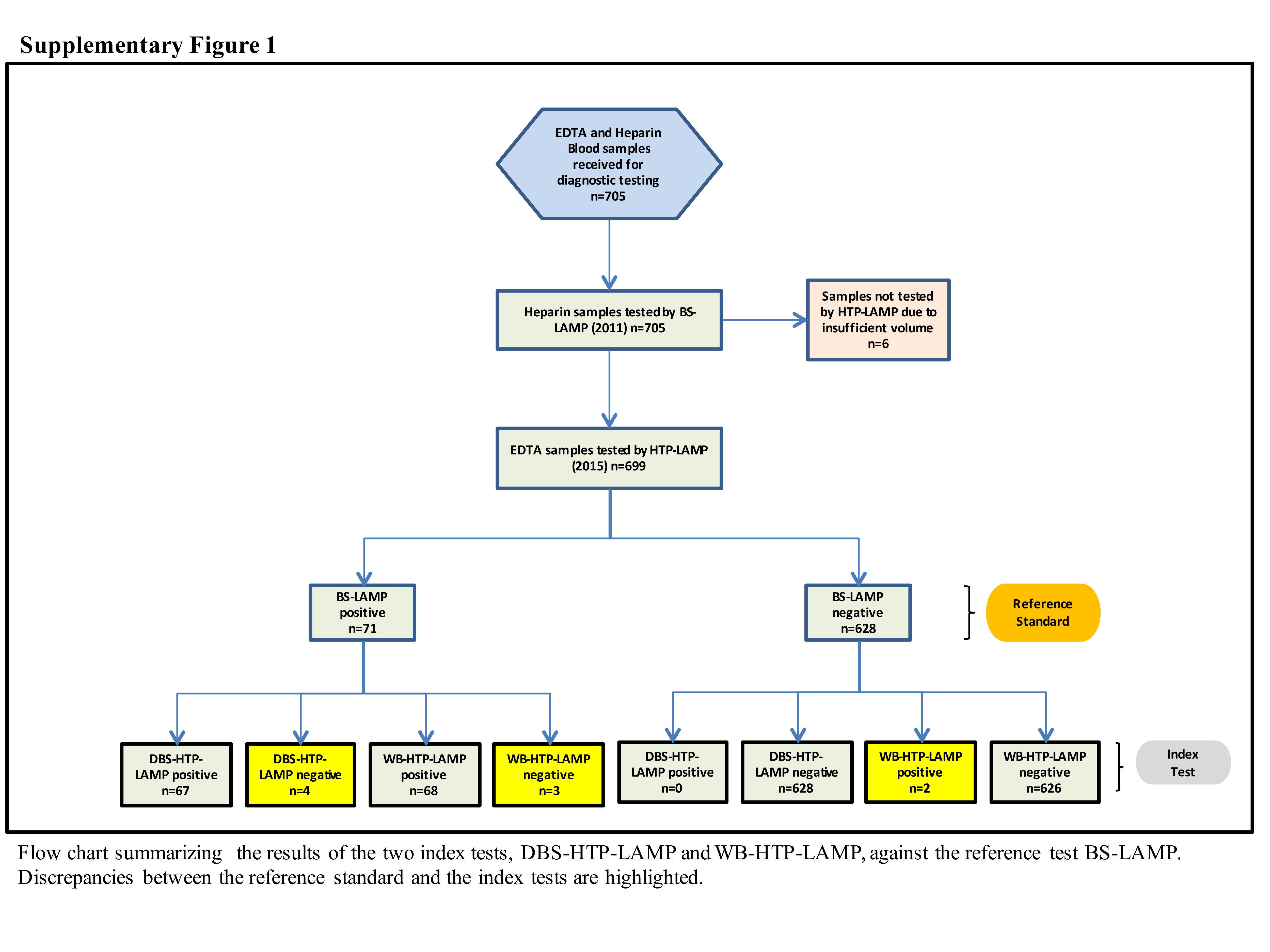

Supplement: S1 Fig — (TIF) [file pone.0171126.s001.tif]

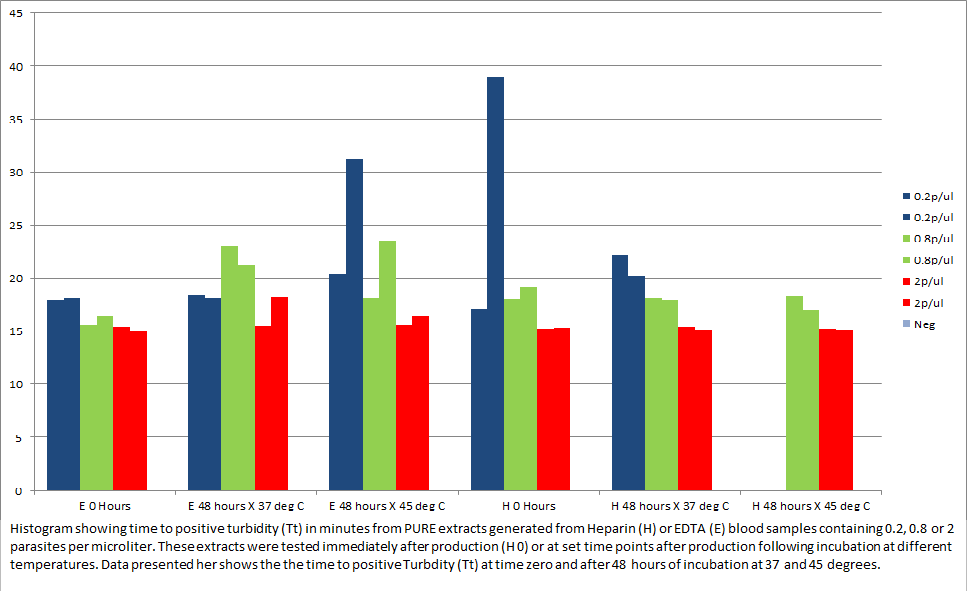

Supplement: S2 Fig — (TIF) [file pone.0171126.s002.tif]
