# Supplementary material for: Development and clinical performance of high throughput loop-mediated isothermal amplification for detection of malaria
Source: PLoS One. 2017 Feb 6;12(2):e0171126. doi: 10.1371/journal.pone.0171126 (PMC5293265; doi:10.1371/journal.pone.0171126)
Supplement: S1 Text — (DOCX) [file pone.0171126.s003.docx]

**Supplementary Information**

Development and Clinical Performance of High Throughput Loop-Mediated Isothermal Amplification for detection of Malaria.

Boil-and-spin DNA extraction

Boil and spin DNA extractions were used for sample quality control purposes and performed according to the FIND “Manual of Standard Operating Procedures for malarial LAMP” [20]. Briefly, 20µL of whole EDTA or heparinised blood was added to 20µL extraction buffer (400mM NaCl, 40mM Tris pH 6.5, 0.4% sodium dodecyl sulphate SDS) in 0.2mL PCR tubes and heated for 5 min at 95 ^0^C in a thermocycler, and cooled to 37 ^0^C for 5 min. The samples were centrifuged at 14,000 x g for 5 min. 2.2µL of clear supernatant was diluted in 27.8µL of PCR water.

Sample quality control

BS-LAMP performed using heparinised blood samples identified 71 positives out of 705 samples [18]. The HTP-LAMP assay was performed on 699 of the original 705 using EDTA samples. In order to ensure that long term storage of the EDTA and heparinised blood samples had not resulted in DNA degradation, BS-LAMP was performed on available EDTA and corresponding heparin blood samples. This comparator test allowed validation of results obtained from the current HTP-LAMP assay for comparison against the original PCR and BS-LAMP results, as well as a comparison between BS-LAMP performed on EDTA and heparin blood samples.

BS-LAMP was performed on available EDTA and corresponding heparin blood samples (n = 62). This set included 60 positives identified by BS-LAMP, which included one sample positive by BS-LAMP and negative by PCR, WB and DBS HTP-LAMP and one sample positive by BS-LAMP and PCR though negative by WB and DBS HTP-LAMP (Table A). In addition, two samples positive by WB-HTP-LAMP, and negative by all other tests were also tested. There was insufficient material to test the remaining 11 positives originally identified by BS-LAMP (Table A).

Out of the 60 positives, 59 heparin blood samples tested positive, while 57 EDTA blood samples tested positive (Table A). This indicates that the majority of samples in both sets survived long-term storage and retained DNA quality. One sample negative for both EDTA and heparin BS-LAMP, was also negative by WB and DBS-HTP-LAMP, though positive by PCR and the original BS-LAMP. This sample had a parasitaemia lower than 1p/µL. It is possible that long-term storage may have caused some degradation of DNA resulting in the false negative result. A sample positive by both heparin BS-LAMP as well as the original heparin BS-LAMP from 2011, was negative by EDTA BS-LAMP, nPCR and WB and DBS HTP-LAMP (Table A). This was a follow up sample from a patient who had tested positive previously, though negative afterwards. Therefore it is likely this was a low density parasitaemia, which was only detected by the original heparin BS-LAMP. One sample negative by both original and current heparin BS-LAMP, negative by nPCR and DBS-HTP-LAMP, was positive by EDTA BS-LAMP and WB-HTP-LAMP. One other sample was negative by all except WB-HTP-LAMP.

**Table A. Reconfirmation of sample quality of 62 EDTA and Heparin blood samples by BS-LAMP.**

|  | Number of Samples | BS-LAMP Heparin 2011 | PCR  (p/µL) | DBS-HTP-LAMP | WB-HTP-  LAMP | BS-LAMP Heparin 2016 | BS-LAMP EDTA 2016 |  |
| --- | --- | --- | --- | --- | --- | --- | --- | --- |
|  | 57 | Positive | Positive  (<1 to 912,896/µL) | Positive | Positive | Positive | Positive |  |
|  | 1 (HL585) | Positive | Positive  (0.5/µL) | Positive | Positive | Positive | Negative |  |
|  | 1 (HL1543) | Positive | Negative | Negative | Negative | Positive | Negative |  |
|  | 1 (HL1273) | Positive | Positive  (0.8/µL) | Negative | Negative | Negative | Negative |  |
|  | 1 (HL1005) | Negative | Negative | Negative | Positive | Negative | Positive |  |
|  | 1 (HL1614) | Negative | Negative | Negative | Positive | Negative | Negative |  |

BS-LAMP vs WB-HTP-LAMP and DBS-HTP-LAMP

When WB-HTP-LAMP was compared to the original BS-LAMP data as an alternative to the gold standard nested PCR, a similar diagnostic accuracy was seen. The WB-HTP-LAMP assay failed to detect 3 of the 71 positive BS-LAMP samples, showing a sensitivity of 95.8% (95% CI, 91.1-100) (Table B). From the three negative WB-HTP-LAMP samples, one sample was positive by PCR. Real-time PCR results showed this sample had a parasite density lower than 1p/µL, which is below the known detection limit for WB-HTP-LAMP. The other two samples were negative by PCR. One of these samples was a follow-up test from a patient who previously tested positive. It is possible the other WB-HTP-LAMP and PCR negative sample presented as positive for BS-LAMP due to contamination. The WB-HTP-LAMP assay detected two positive samples which were BS-LAMP negative, giving a specificity of 99.7% (95% CI, 99.2-100) (Table B). It is possible the two false positive results are due to contamination.

By comparison, the DBS-HTP-LAMP assay failed to detect 4 of the 71 positive BS-LAMP samples, giving a sensitivity of 94.4% (95% CI, 89-99.7) (Table B). Two of the 4 samples positive by BS-LAMP were negative by nPCR, while two samples positive by both BS-LAMP and nPCR showed parasites densities lower than 1p/µL. It is also possible that samples found positive only by BS-LAMP may be due to the occurrence of contamination leading to false positive results. The DBS-HTP-LAMP assay accurately detected all BS-LAMP negative samples giving a specificity of 100% (Table B).

Compared with BS-LAMP, the positive predictive value (PPV) of DBS-HTP-LAMP and WB-HTP-LAMP was 100% and 97.1% respectively. The negative predictive value (NPV) of DBS-HTP-LAMP was 99.4% and WB-HTP-LAMP was 99.5% (Table B).

A pairwise comparison between BS-LAMP and WB-HTP-LAMP gave a P value of 0.6547 indicating statistical equivalence of WB-HTP-LAMP to BS-LAMP. However, the pairwise comparison between BS-LAMP and DBS-HTP-LAMP gave a P value of 0.045 indicating the statistical non-equivalence of DBS-HTP-LAMP to BS-LAMP (Table B).

**Table B. Comparison of Malaria HTP-LAMP results from 699 DBS and WB samples against the BS-LAMP results from 2011.**

|  |  |  |  |  |  |  |  |  |  |
| --- | --- | --- | --- | --- | --- | --- | --- | --- | --- |
|  | **Comparison** | **BS-LAMP (+)** | **BS-LAMP (-)** | **Sensitivity**  **(95% CI)** | **Specificity (95% CI)** | **PPV, %** | **NPV, %** | **Test for non-inferiority** |  |
|  | **DBS-HTP-LAMP vs. BS-LAMP**  **n = 699** |  |  |  |  |  |  |  |  |
|  | DBS HTP-LAMP (+) | 67 | 0 | 94.4 | 100 | 100 | 99.4 | P = 0.0455 |  |
|  | DBS HTP-LAMP (-) | 4 | 628 | (89-99.7) |  |  |  |  |  |
|  |  |  |  |  |  |  |  |  |  |
|  | **WB-HTP-LAMP vs. BS-LAMP**  **n = 699** |  |  |  |  |  |  |  |  |
|  | WB-HTP-LAMP (+) | 68 | 2 | 95.8 | 99.7 | 97.1 | 99.5 | P = 0.6547 |  |
|  | WB-HTP-LAMP (-) | 3 | 626 | (91.1-100) | (99.2-100) |  |  |  |  |
|  |  |  |  |  |  |  |  |  |  |

The McNemar test, used to conduct a pairwise comparison between boil and spin LAMP and index text WB-HTP-LAMP, showed statistical equivalence

between both tests (P=0.6547), while the DBS-LAMP index test was found statistically non-equivalent to the reference BS-LAMP (P=0.0455).

**Table C. Comparison of various high throughput LAMP platforms**

| **LAMP Platform** | **References** | **Advantages** | **Disadvantages** |
| --- | --- | --- | --- |
| **Colorimetric LAMP using Hydroxynaphthol Blue** | **21, 22** | - **Use of 96 well plates for high throughput LAMP detection of malaria parasites** - **Visual end point high throughput detection of positive and negative results** | - **Time taken for sample processing** - **Separate sample processing steps** - **Use of liquid reagents requiring cold storage and extra pipetting steps** - **Turnaround time from sample preparation to reading results 4-6 hours** |
| **Colorimetric LAMP using Malachite Green** | **23** | - **Compatible with use of DBS and WB** - **Visual end point high throughput detection of positive and negative results** | - **Time taken for sample processing** - **Separate sample processing steps** - **Use of liquid reagents requiring cold storage and extra pipetting steps** |
| **Illumigene LAMP** | **24** | - **Compatible with use of DBS and WB** - **Field stable, pre-dispensed lyophlised LAMP reagents** - **Turnaround time <1 hour from sample preparation to reading results** | - **Low throughput, 10 samples processed in <1 hour** |
| **HTP-LAMP** |  | - **Compatible with use of DBS and WB** - **Field stable, pre-dispensed lyophlised LAMP reagents** - **Turnaround time <2 hours from sample preparation to results for 96 samples** | - **Electricity requirement** |
